# Accounting for serotypic heterogeneity improves the interpretation of pneumococcal vaccine response using serotype-specific ELISA IgG concentrations

**DOI:** 10.1128/spectrum.00661-26

**Published:** 2026-09-11

**Authors:** Gatien Durand, Camillia Belhoul, Merieme Bensalah, Maxime Jeljeli, Laurie Toullec, Marine Gil, Elodie Lachiche, Camille Baron, Claire Goulvestre, Christian Drouet, Frédéric Batteux, Lucie Chevrier

**Affiliations:** 1 Université Paris Cité, Faculté de Médecine, AP-HP-Centre Université de Paris, Hôpital Cochin, Service d’immunologie biologique, Paris, France; 2 Département 3I Infection, Immunité et Inflammation, Institut Cochin, INSERM U1016, Université Paris Cité, Paris, France; 3 Université Grenoble Alpes 27015, Saint-Martin-d'Hères, Auvergne-Rhône-Alpes, France; 4 Institut Pasteur de Lille 56099, Lille, France; Naturwissenschaftliches und Medizinisches Institut an der Universitat Tubingen, Reutlingen, Germany

**Keywords:** pneumococcal vaccine, ELISA, OPA, heterogeneity, serotypes

## Abstract

Assessment of pneumococcal vaccine response by enzyme-linked immunosorbent assay (ELISA) currently relies on a single IgG concentration threshold applied to all serotypes. This approach does not account for the marked serotype variability in the relationship between IgG concentration and functional antibody activity. We investigated whether accounting for serotypic heterogeneity improves the interpretation of pneumococcal vaccine response by establishing serotype-specific IgG concentrations predictive of a positive OPA response (PIC). We analyzed 729 samples from adults referred for assessment of pneumococcal vaccine response. For seven vaccine serotypes, IgG concentrations were measured by ELISA and functional activity of antibodies was assessed by opsonophagocytic assay (OPA). IgG concentrations differed significantly according to both serotype and OPA status. Quantitative analyses demonstrated significant correlations between IgG concentrations and OPA titers for all serotypes (Spearman’s ρ = 0.45–0.76; all *P* < 0.0001). For each serotype, PIC was determined using receiver operating characteristic (ROC) curve analysis, Youden’s index, and bootstrap validation. PIC values ranged from 0.84 to 4.74 µg/mL and differed significantly between serotypes (*P* < 0.0001). Diagnostic performance is good, with areas under the curve ranging from 0.73 to 0.87. Application of serotype-specific PICs significantly modified vaccine response classification compared with the use of a single universal threshold (Cochran’s Q and McNemar’s tests, *P* < 0.0001). Accounting for serotypic heterogeneity improves the interpretation of pneumococcal ELISA results by providing PIC that more closely reflect functional OPA responses. Although validation in clinically well-characterized patient cohorts remains necessary, this approach may facilitate interpretation of vaccine responses when OPA is not feasible.

**IMPORTANCE:**

In routine clinical practice, pneumococcal vaccine responses are frequently assessed using serotype-specific IgG concentrations measured by enzyme-linked immunosorbent assay (ELISA), commonly interpreted with a single IgG cut-off applied uniformly across serotypes. However, this approach does not account for serotypic variability. Furthermore, it assumes that identical IgG concentrations reflect comparable functional antibody activity across serotypes. Opsonophagocytosis testing can determine antibody functionality, but these tests are difficult to implement routinely in clinical laboratories. Considering, on the one hand, the heterogeneity of the vaccine response and, on the other hand, the relationship between antibody concentration and functionality could help in evaluating the vaccine response in patients at risk of invasive pneumococcal disease.

## INTRODUCTION

*Streptococcus pneumoniae* remains a major cause of invasive bacterial infections worldwide, particularly among adults with comorbidities or immune dysfunction (1). Despite widespread vaccine implementation, invasive pneumococcal disease (IPD) still occurs in vaccinated individuals (2, 3), underscoring the need for accurate laboratory tools to assess vaccine-induced immunity at the individual level (4).

In clinical practice, evaluation of pneumococcal vaccine responses commonly relies on serotype-specific IgG concentrations measured by enzyme-linked immunosorbent assay (ELISA) (5), interpreted using a single IgG cut-off applied uniformly across serotypes. Although these cut-offs were initially developed for population-level vaccine evaluation and licensure frameworks, they have been widely adopted for individual patient management in immunology and clinical microbiology laboratories (6, 7). However, this approach assumes that identical ELISA IgG concentrations reflect comparable functional antibody activity across all serotypes, despite evidence that the quantitative relationship between ELISA-measured antibodies and opsonophagocytic activity varies substantially according to serotype.

Functional antibody activity against pneumococcus can be assessed using the opsonophagocytic assay (OPA), which measures the capacity of antibodies to promote complement-mediated bacterial killing (8, 9). OPA is widely regarded as a functional reference assay for pneumococcal immunity, but it remains technically demanding, time-consuming, and not routinely available in many laboratories. Consequently, understanding how ELISA IgG concentrations quantitatively relate to functional OPA responses is essential for interpreting routine pneumococcal serology. As a result, ELISA is typically used as a first-line assay, while OPA is reserved for selected clinical situations, such as inconclusive serological profiles or evaluation of immunocompromised patients (10).

Multiple lines of evidence support substantial serotype-dependent heterogeneity in pneumococcal immune responses, including differences in baseline IgG concentrations, vaccine immunogenicity, and functional antibody activity (11, 12). These observations call into question the relevance of a universal ELISA cut-off for interpreting pneumococcal serology in individual patients. Rather than proposing universal protective IgG thresholds, a more informative approach is to characterize, for each serotype, the quantitative relationship between ELISA IgG concentrations and functional OPA responses. Such an approach could improve interpretation of ELISA results when routine OPA testing is not feasible, while identifying situations in which functional testing remains warranted.

Here, we performed a retrospective analysis of 729 serum samples from adult patients evaluated for pneumococcal vaccine responses in routine clinical practice. For seven pneumococcal vaccine serotypes, we compared serotype-specific IgG concentrations measured by ELISA with corresponding OPA results. Using receiver operating characteristic (ROC) analysis, Spearman correlation, linear regression, and bootstrap resampling, we characterized the quantitative relationship between ELISA IgG concentrations and OPA titers and estimated serotype-specific IgG concentrations predictive of a positive OPA response (PIC). Our objective was not to define protective cut-offs for clinical decision-making but to describe serotype-dependent heterogeneity in the ELISA–OPA relationship and to assess its impact on pneumococcal serology interpretation.

By delineating serotype-specific variability in IgG concentrations associated with functional opsonophagocytic activity, this work aims to improve interpretability of pneumococcal serological testing and to support a more rational, targeted use of OPA in clinical immunology and microbiology laboratories.

## MATERIALS AND METHODS

### Samples

Between January 2018 and June 2024, 729 serum samples from adult patients were referred to the Department of Biological Immunology (Cochin Hospital, Assistance Publique—Hôpitaux de Paris) for evaluation of pneumococcal vaccine response. ELISA and OPA were performed for the seven serotypes included in the seven-valent pneumococcal conjugate vaccine (PCV7: 4, 6B, 9V, 14, 18C, 19F, and 23F). These investigations were performed to determine whether patients had developed an adequate functional immune response following pneumococcal vaccination. According to current recommendations, an adequate vaccine response was defined as protective responses against more than 70% of the serotypes tested. Data were collected in accordance with the regulations of Assistance Publique—Hôpitaux de Paris. Because this was a retrospective study based on fully anonymized laboratory data, no identifiable patient information was available. Consequently, demographic characteristics, underlying clinical conditions, immunosuppressive treatments, vaccination history, and time since vaccination could not be assessed.

### Enzyme-linked immunosorbent assay

Polysaccharide-specific IgG antibody concentrations were measured by ELISA following analytical WHO recommendations (www.vaccine.uab.edu) as previously described (13). Briefly, pneumococcus polysaccharides (SSI Diagnostica, Hillerød, Denmark) were coated in 96-well plates (Greiner Bio-One, Les Ulis, France). Samples, controls, and standard 007-sp (University of Alabama, AL, USA) were pre-absorbed with 5 μg/mL pneumococcal cell wall polysaccharide (SSI Diagnostica) and with 10 μg/mL serotype 22F polysaccharide (SSI Diagnostica), then incubated for 2 h in coated plates. After washing, plates were incubated with alkaline phosphatase-conjugated anti-human goat IgG antibody (SouthernBiotech, AL, USA) for 2 h at room temperature. IgG antibodies were detected with *p*-nitrophenyl phosphate substrate (Euromedex, Souffelweyersheim, France). The absorbance was read at 405 nm by a Multiskan FC spectrophotometer (ThermoFisher Scientific, Asnières-sur-Seine, France). Samples were serially diluted with a twofold dilution from 1/500 to 1/64,000. Anti-pneumococcus antibody concentrations were determined as the mean of diluted concentrations using the four-parameter logistic method compared with the reference sample 007sp (14).

### Opsonophagocytic assay

OPA was performed for seven specific serotypes from the PCV-7 (4, 6B, 9V, 14, 18C, 19F, and 23F) to determine functional antibody responses and was measured according to the WHO-recommended method (www.vaccine.uab.edu), as previously described (13). Briefly, all serum samples were heat-decomplemented and submitted for their spontaneous bactericidal effect prior to OPA. In case of bactericidal effect, the sample was discarded. Serum samples were serially diluted in round-bottom 96-well plates (Corning, Boulogne-Billancourt, France) and incubated with bacteria (~50,000 colony-forming units [CFU]/mL) for 30 min at room temperature. Complement (Gentaur France, Paris, France) and HL60 cells (ATCC, VA, USA) that had been differentiated to phagocytes (0.5 million) were added to each well. After a 45 min incubation (37°C, 5% CO_2_, 700 rpm), the plates were incubated on ice for 20 min. Bacteria from each well were spotted onto Todd-Hewitt broth-yeast extract agar plates. After an overnight incubation at 37°C, the number of CFU in the plates was enumerated using NICE® software (NIST’s Integrated Colony Enumerator) (https://www.nist.gov/publications/low-cost-high-throughput-automated-counting-bacterial-colonies). For each sample, the opsonization index (OI) was calculated as the interpolated reciprocal serum dilution resulting in 50% bacterial killing, providing a continuous quantitative measure of functional antibody activity. For diagnostic performance analyses, OI values were subsequently classified as positive or negative using the predefined serotype-specific OPA positivity criteria. The assay sensitivity is the lowest dilution of sera tested (limit of detection), which is usually 4 for undiluted sera, and the same for each serotype.

The bacterial strains were obtained in the laboratory of Prof. Moon H. Nahm (WHO pneumococcal serology reference laboratory, University of Alabama).

### ROC curve and Youden index

For each pneumococcus serotype, in order to determine the optimal PIC, we used the ROC curve method. The samples were categorized according to the OPA result: positive if greater than or equal to the positivity threshold and negative if less than the positivity threshold. For each sample, we calculated sensitivity and specificity, then plotted the ROC curve (sensitivity on the *y*-axis, 1 – specificity on the *x*-axis) (15). PIC can be determined using the Youden J index (J = sensitivity + specificity − 1). It is calculated for each sample, the highest index corresponding to the PIC. The coefficient of variation of the concentration was also calculated.

### Bootstrap

Analysis using ROC curves and the Youden index generates a PIC single value for each serotype, which does not allow for statistical testing. To overcome this limitation, we used the bootstrap method. This approach involves generating new samples by randomly drawing samples from the cohort with replacement, then recalculating the PIC at each time. Repeating a defined number of times per serotype, this process yields a PIC distribution that reflects the variability of the estimate. For each serotype, we applied bootstrapping with replacement, performing 10, 100, 1,000, and 10,000 resamplings of the same size as the initial cohort. We chose the threshold of 1,000 resamplings as the best compromise between statistical power and biological relevance for analyzing PIC heterogeneity for each serotype.

### Normality test

Shapiro-Wilk test did not validate the normality of the distribution of specific Ig concentration values by ELISA, which led us to use non-parametric tests.

### Kruskal–Wallis and Dunn’s post hoc test with Holm correction

PIC determined by bootstrap was non-normally distributed for all serotypes (Shapiro-Wilk, *P* < 0.0001); non-parametric methods were performed for group comparisons. Overall heterogeneity across serotypes was assessed according to the Kruskal–Wallis test and, in cases of significant results, we applied a Dunn’s post hoc test with Holm correction to identify pairwise differences for multiple testing. Holm-adjusted *P* values < 0.05 were considered statistically significant.

### Correlation and regression analyses

The quantitative relationship between ELISA IgG concentrations and OPA titers was assessed separately for each serotype using Spearman’s rank correlation coefficient. Correlation analyses were performed on paired ELISA and OPA measurements, and two-sided *P* values were calculated to test the null hypothesis of no monotonic association (rho = 0). Correlation coefficients and associated *P* values are reported in Table S1.

### Statistical analysis

All statistical tests were performed using Python software V3.10 (Wilmington, DE, United States) with the following plugins: Pandas V1.5.3, Sklearn, matplotlib 3.6.3, Scipy 1.10.1, Scikit-learn 1.2.1, Scikit-posthocs 0.11.4, Statsmodels 0.14.4, and Numpy 1.24.1. The applied test was introduced in the legends of the figures and tables.

All analyses were exploratory in nature. PIC were estimated using internal bootstrap resampling to assess robustness, but no external validation cohort was available. Results should therefore be interpreted as hypothesis-generating and intended to inform test interpretation rather than to define clinical decision thresholds.

## RESULTS

### Cohort description

Between January 2018 and June 2024, 729 serum samples from adult patients were registered to perform an ELISA and an OPA directed against the seven serotypes of PCV-7 (4, 6B, 9V, 14, 18C, 19F, and 23F). The purpose of these explorations was to determine whether a patient had sufficient vaccine protection at a given time. Before July 2023, French vaccination recommendations were as follows: one dose of PCV13, then boost vaccination with PPV23. After July 2023, a unique dose of PVC20 is recommended.

The median IgG concentration varied markedly across serotypes, ranging from 0.67 μg/mL for the lowest (serotype 4) to 5.17 μg/mL for the highest (serotype 14) (Table 1), suggesting substantial serotype-dependent variability in quantitative antibody responses.

**TABLE 1:** Median concentration of vaccine serotype-specific IgG and variability in a series of 729 patient samples^*a*^

Pneumococcus serotype	ELISA IgG concentration (µg/mL)	
	Median	95% CI
4	0.67	0.13–4.85
6B	1.93	0.32–18.05
9V	1.00	0.12–9.10
14	5.17	0.54–42.05
18C	1.38	0.20–11.23
19F	2.80	0.48–21.85
23F	1.30	0.17–17.28

^*a*^ CI, confidence intervals; all serotype-specific ELISAs were performed on the 729 samples.

**Fig 1:**
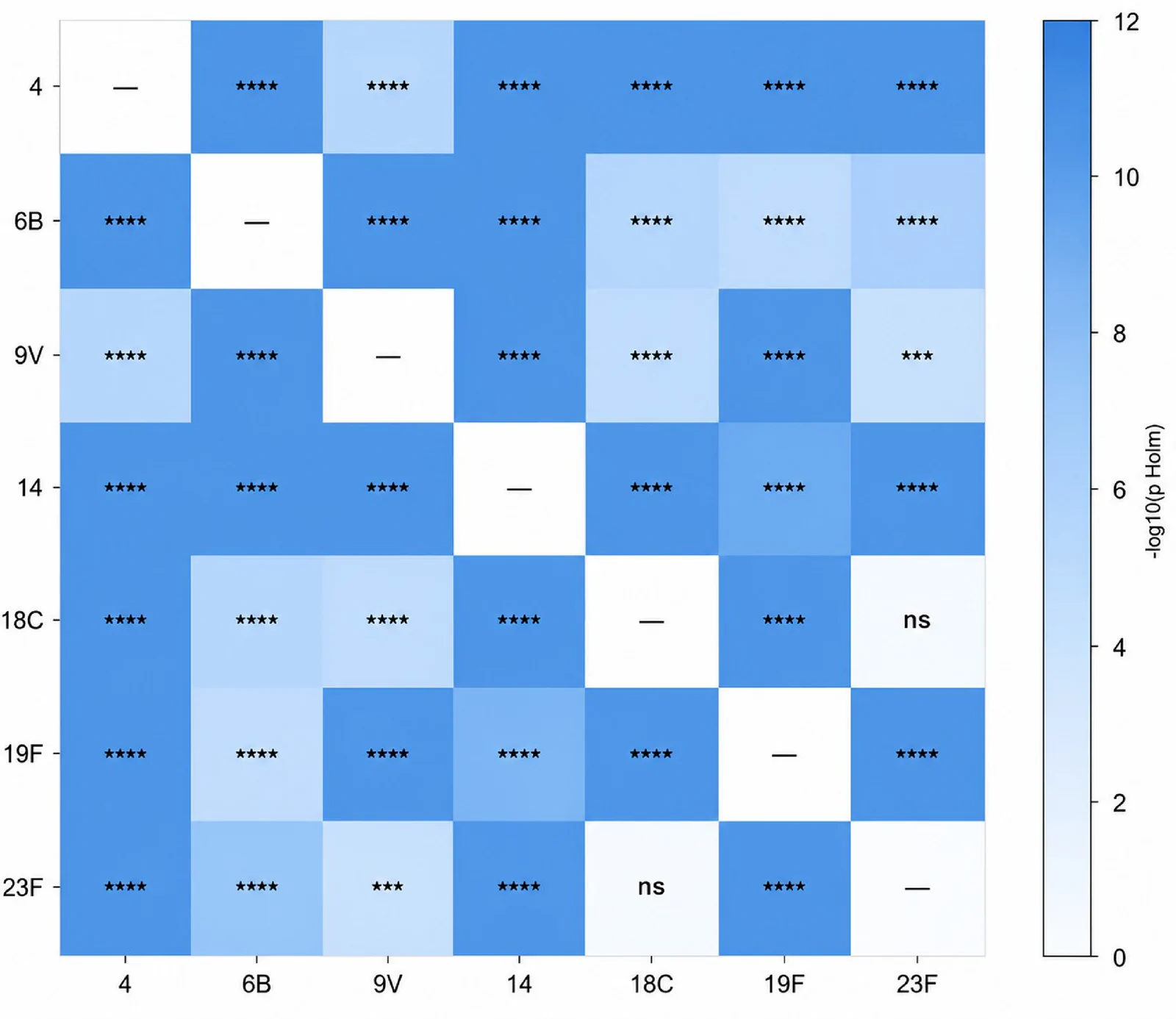
Serotype-dependent heterogeneity of anti-pneumococcal IgG responses. Heatmap representing pairwise adjusted *P* values obtained from Dunn’s post hoc comparisons of serotype-specific ELISA IgG concentrations following Kruskal–Wallis analysis. Color intensity corresponds to −log10(adjusted *P* value). Statistical significance is indicated as ns (not significant), *P* < 0.05 (*), *P* < 0.01 (**), *P* < 0.001 (***), and *P* < 0.0001 (****).

The IgG concentrations between serotypes were then compared by applying a non-parametric Kruskal–Wallis test. We highlighted a heterogeneity of serotype-specific immune response [H(6) = 1,017, *P* < 0.0001, where H corresponds to the Kruskal–Wallis test statistic with 6 degrees of freedom]. Due to this high degree of heterogeneity observed between serotypes, we performed a post hoc Dunn test with Holm correction to identify significant differences between serotypes. Except between serotypes 18C and 23F, where no significant differences were observed, pairwise comparison of the antibody concentration heterogeneity was demonstrated between the other serotypes. All data are shown in Fig. 1.

### Determining serotype-specific predictive IgG concentration

OPA results were displayed in a binary distribution: an OI higher than the positivity threshold indicated the production of functional antibodies against the tested serotype. Conversely, an OI lower than the positivity threshold highlighted a defective functionality of the antibodies. For patients with a positive OPA, the lowest IgG concentration was 1.06 μg/mL for serotype 4, and the highest IgG concentration was 10.24 μg/mL for serotype 14 (Table 2). For patients with a negative OPA, data ranged from 0.46 to 2.26 μg/mL for serotypes 4 and 14, respectively.

**TABLE 2:** Distribution of anti-polysaccharide IgG concentrations according to OPA test on serum samples^*b*^

Pneumococcus serotype	IgG median concentration (µg/mL) (95% CI)		
	OPA negative test	OPA positive test	*P* value^*a*^
4	0.46 (0.11–2.09) (*n* = 365)	1.06 (0.20–6.42) (*n* = 364)	<0.0001
6B	1.32 (0.22–4.47) (*n* = 333)	3.12 (0.60–28.48) (*n* = 396)	<0.0001
9V	0.64 (0.10–3.10) (*n* = 358)	1.80 (0.28–12.71) (*n* = 371)	<0.0001
14	2.26 (0.30–11.15) (*n* = 332)	10.24 (2.28–73.71) (*n* = 397)	<0.0001
18C	0.75 (0.15–3.34) (*n* = 336)	2.51 (0.55–15.75) (*n* = 393)	<0.0001
19F	1.50 (0.28–6.98) (*n* = 226)	3.54 (0.66–28.24) (*n* = 503)	<0.0001
23F	0.73 (0.14–3.83) (*n* = 411)	3.71 (0.40–36.67) (*n* = 318)	<0.0001

^*a*^ Calculated using Mann–Whitney tests.

^*b*^ CI, confidence intervals; *n*, number of patients.

The evidence of heterogeneity prompted us to determine the allocation of IgG concentrations for each serotype. Based on the binary distribution of the OPA results, we next determined for each serotype an IgG concentration that could be predictive of functional antibody activity (i.e., positive OPA result), named PIC for predictive IgG concentration.

**Fig 2:**
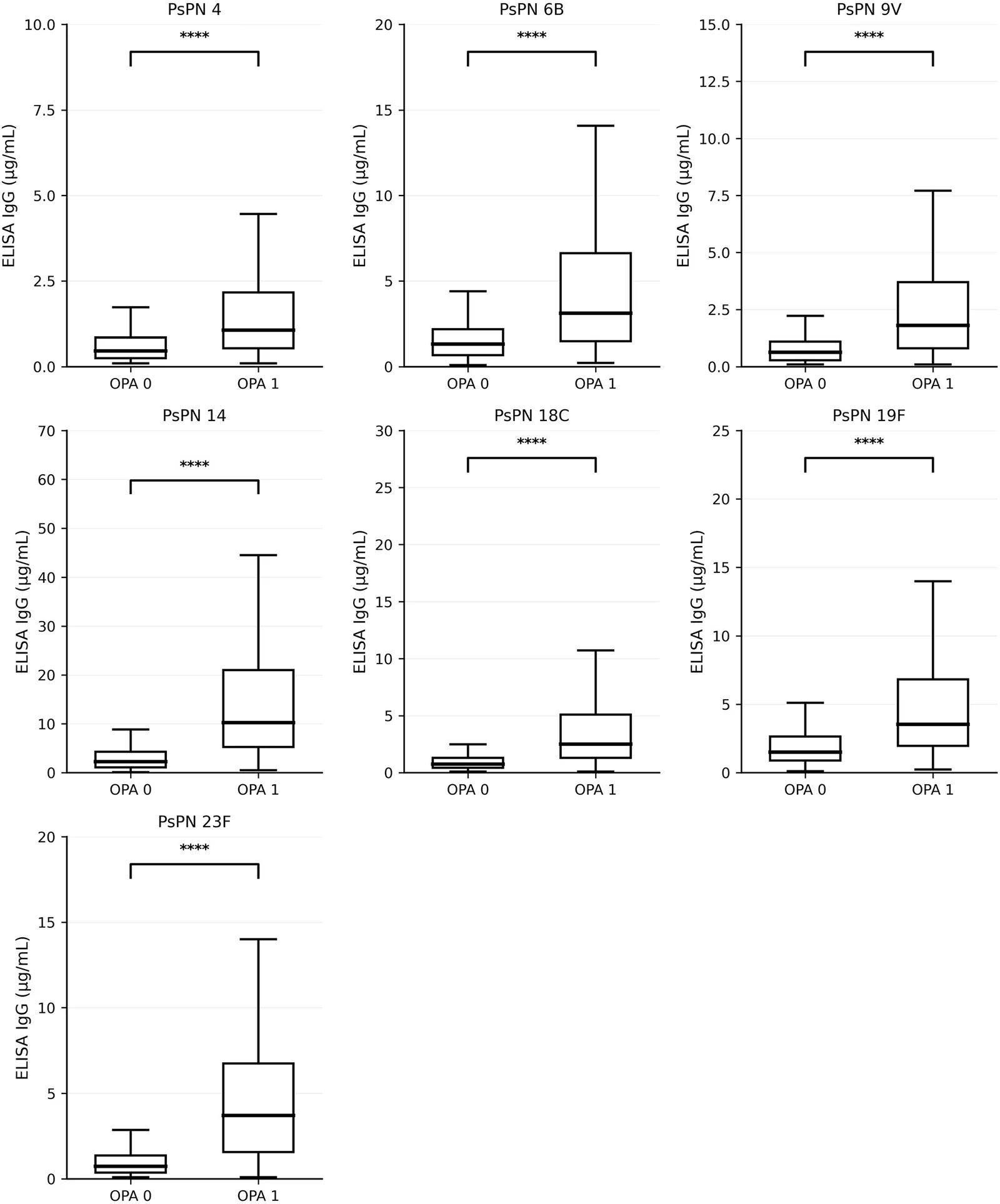
Relationship between ELISA IgG concentrations and OPA outcome. Anti-pneumococcal ELISA IgG concentrations were compared between OPA-positive (OPA = 1) and OPA-negative (OPA = 0) samples for each serotype. Boxes represent the interquartile range (IQR), center lines indicate the median, and whiskers indicate 1.5 × IQR. Group differences were assessed using a Mann–Whitney test. ****P < 0.0001.

First, we compared IgG concentration between groups with OPA-positive and groups with OPA-negative results (Table 2, Fig. 2). For all serotypes, a higher concentration in ELISA was found for OPA-positive results. Due to the observation of a wide variation in median IgG concentrations between the different serotypes, we wanted to determine whether there was a significant difference between them. We performed a Kruskal–Wallis test for ELISA concentrations associated with a negative OPA result [H(6) = 496, *P* < 0.0001 where H corresponds to the Kruskal–Wallis test statistic with 6 degrees of freedom] and for ELISA concentrations with a positive OPA result [H(6) = 697, *P* < 0.0001 where H corresponds to the Kruskal–Wallis test statistic with 6 degrees of freedom], confirming marked serotype-dependent heterogeneity in both groups.

**Fig 3:**
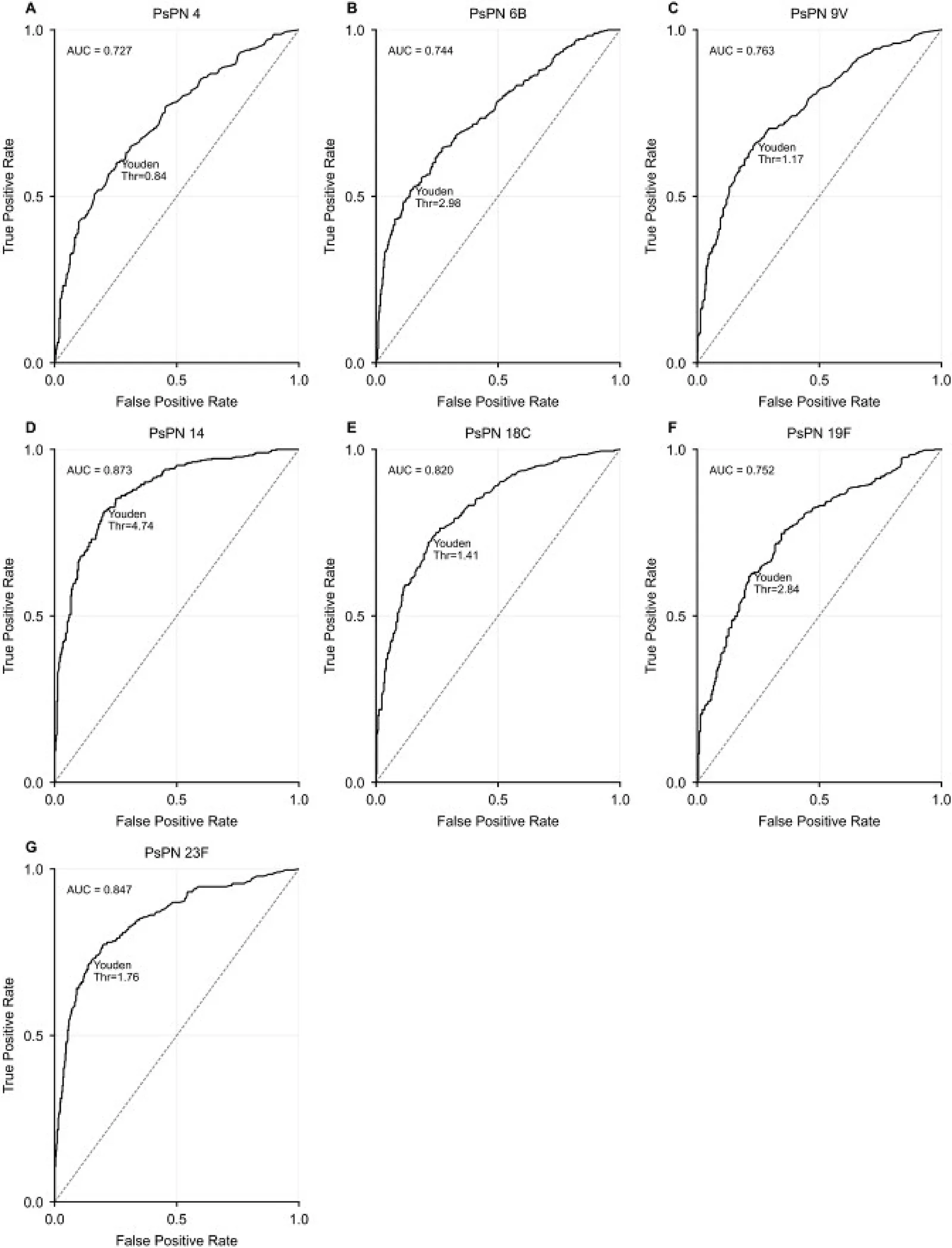
ROC analysis of ELISA IgG concentrations predictive (PIC) of OPA positivity. ROC curves evaluating the performance of serotype-specific ELISA IgG concentrations predictive of OPA positivity (PIC) were determined using the Youden index. AUCs are indicated for each serotype. Panels **A–G** correspond to serotypes PsPN4, 6B, 9V, 14, 18C, 19F, and 23F.

Next, we performed an ROC curve analysis based on the binary and discriminating nature of the OPA result to determine the PIC (Fig. 3). For each ROC curve, the area under the curve (AUC) shows at least an acceptable analytical performance (AUC > 0.70) or even an excellent ability to discriminate a positive response by OPA (AUC > 0.80).

In order to obtain the most accurate estimate of the PIC, the Youden index method was applied. The concentration calculation method using Youden’s J index (J = sensitivity + specificity − 1) for each serotype offers the best compromise between sensitivity and specificity. PIC varied between serotypes, ranging from 0.84 μg/mL for serotype 4 to 4.74 μg/mL for serotype 14, further confirming the marked serotype-dependent heterogeneity observed in the previous analyses (Table 3).

**TABLE 3:** Predictive IgG concentrations of functional antibodies determined using ROC curves and Youden’s index^*a*^

Pneumococcal serotype	Predictive IgG concentration of functional antibody (PIC) µg/mL (95% CI)	Se	Sp	AUC
4	0.84 (0.19–7.20)	0.60	0.75	0.73
6B	2.98 (0.32–9.03)	0.52	0.86	0.74
9V	1.17 (0.21–6.63)	0.65	0.77	0.76
14	4.74 (0.66–23.49)	0.81	0.80	0.87
18C	1.41 (0.26–8.16)	0.72	0.79	0.82
19F	2.84 (0.46–14.46)	0.62	0.79	0.75
23F	1.79 (0.23–10.48)	0.71	0.86	0.85

^*a*^ CI, confidence intervals; Se, sensitivity; Sp, specificity; AUC, area under the curve.

### Bootstrap analysis and evaluation of heterogeneity

To assess the robustness of PIC estimates, we performed a bootstrap analysis with 1,000 resampling iterations. For each iteration, ROC analysis was repeated, and the optimal PIC was recalculated using Youden’s index. The bootstrap-derived median PICs were close to the original estimates, supporting the stability of the thresholds. Median PIC values differed markedly between serotypes, ranging from 0.88 µg/mL for serotype 4 to 4.71 µg/mL for serotype 14 (Table 4).

**TABLE 4:** Median PIC determined after 1,000 bootstrap resampling^*a*^

Serotype	Median PIC (µg/mL)	
	Median	95% CI
4	0.88	0.51–1.32
6B	2.84	1.85–3.47
9V	1.20	0.99–1.74
14	4.71	4.08–6.70
18C	1.38	1.26–2.13
19F	2.78	1.87–3.08
23F	1.68	1.42–2.47

^*a*^ PIC, predictive IgG concentration; CI, confidence intervals.

**Fig 4:**
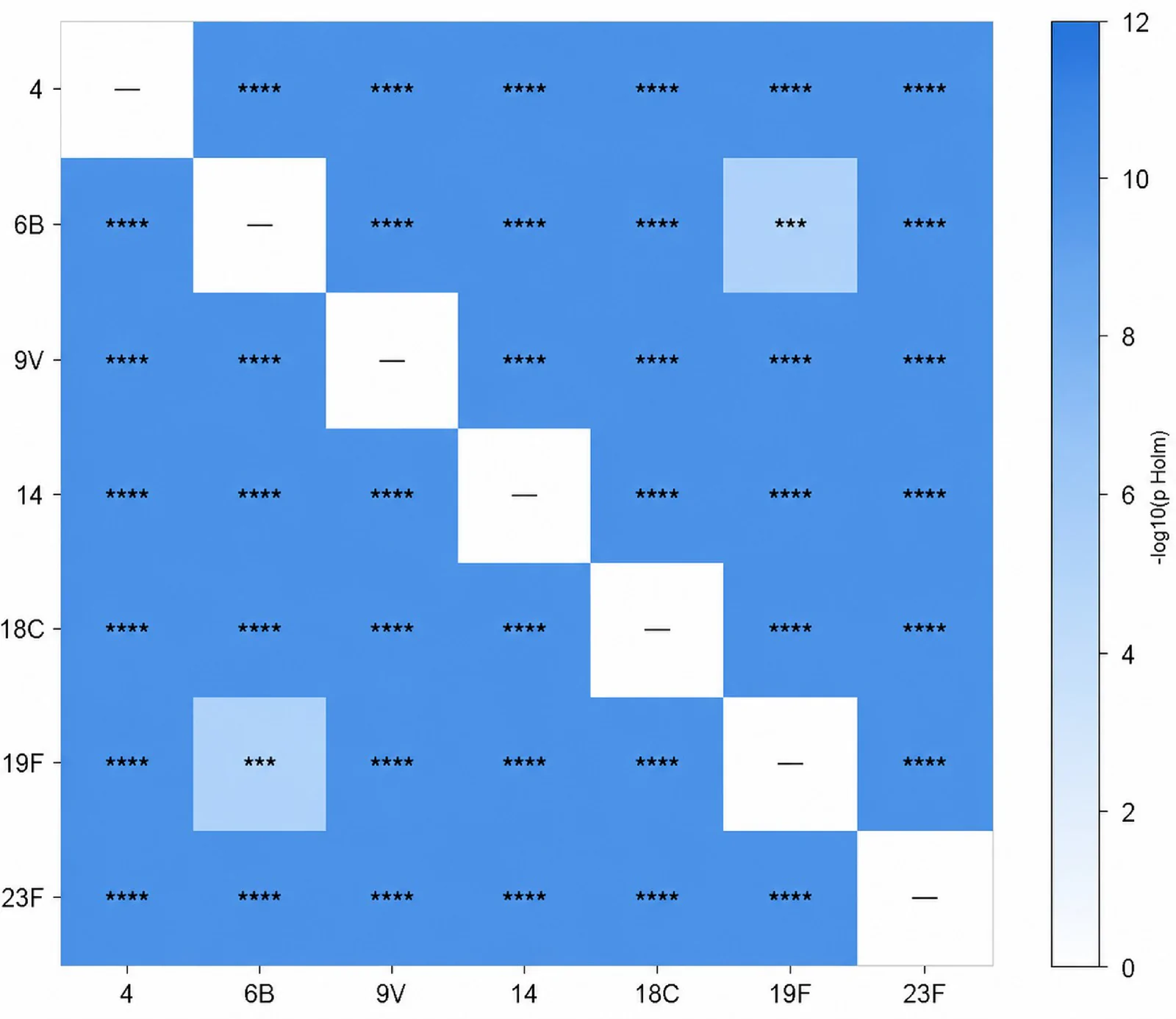
Heterogeneity of serotype-specific predictive IgG concentrations determined by bootstrap analysis. Heatmap showing pairwise comparisons of bootstrap-derived PIC using Dunn's post hoc test following a significant Kruskal–Wallis test. Pairwise comparisons were performed after Holm correction. Statistical significance is indicated as ns (not significant), *P* < 0.05 (*), *P* < 0.01 (**), *P* < 0.001 (***), and *P* < 0.0001 (****).

To formally assess serotype-dependent heterogeneity, bootstrap-derived PIC distributions were compared using the Kruskal–Wallis test. This analysis demonstrated a strong overall difference between serotypes [H(6) = 6,398, *P* < 0.0001, where H corresponds to the Kruskal–Wallis test statistic with 6 degrees of freedom]. Post hoc pairwise comparisons using Dunn’s test with Holm correction confirmed significant heterogeneity between all serotype pairs (Fig. 4). In particular, PIC differed significantly between serotypes 6B and 9V (adjusted *P* value = 0.00004), while most other pairwise comparisons demonstrated even stronger differences (adjusted *P* value < 0.0001), supporting substantial serotype-dependent variability in PIC thresholds.

**Fig 5:**
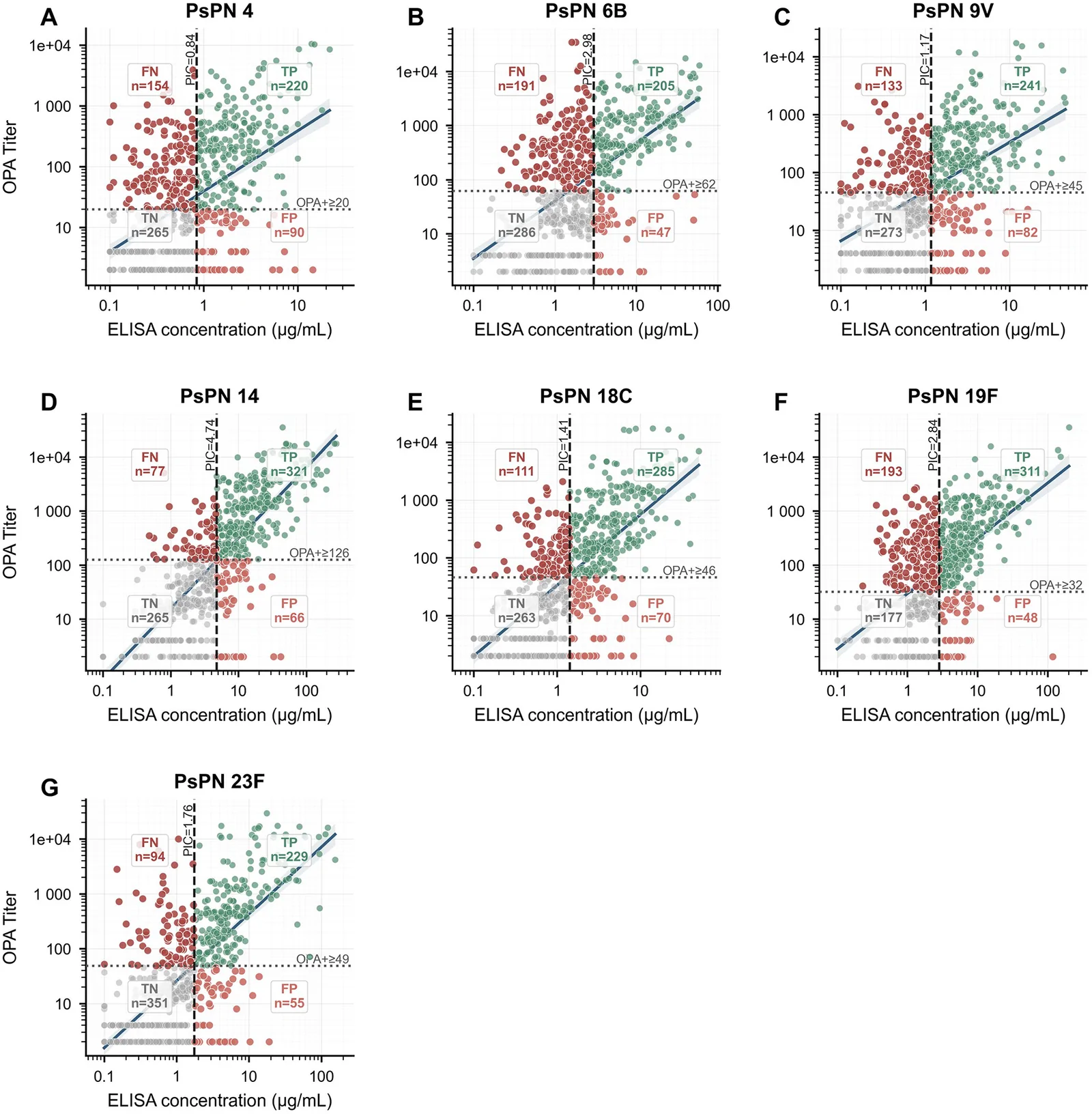
Quantitative relationship between ELISA IgG concentrations and functional OPA titers for each pneumococcal polysaccharide (PsPN). Scatter plots illustrate the relationship between serotype-specific ELISA IgG concentrations (µg/mL) and OPA titers for PsPN 4 (**A**), 6B (**B**), 9V (**C**), 14 (**D**), 18C (**E**), 19F (**F**), and 23F (**G**). Both axes are displayed on a logarithmic scale. The solid blue line represents the linear regression fitted to the log10-transformed data. The vertical dashed line indicates the serotype-specific PIC determined by ROC analysis using Youden's index, whereas the horizontal dotted line indicates the serotype-specific OPA positivity threshold. Samples are classified into true-positive (TP), true-negative (TN), false-positive (FP), and false-negative (FN) quadrants according to the ELISA PIC and OPA positivity threshold.

To better understand the quantitative link between ELISA IgG levels and functional OPA responses, we examined the paired ELISA and OPA data for each serotype individually (Fig. 5). All seven serotypes showed significant positive correlations, with Spearman’s rank correlation coefficients between 0.452 and 0.758 (all *P* < 0.0001; Table S1). After applying a log10 transformation to the ELISA IgG concentrations and OPA titers, linear regression analysis revealed significant quantitative relationships for all serotypes. However, the strength of the association and the regression parameters differed among the serotypes (see Table S2). The sensitivity, specificity, positive predictive value, negative predictive value, and overall accuracy of the serotype-specific PICs are summarized in Table S3. These findings further support the existence of marked serotype-dependent differences in the relationship between ELISA-measured IgG concentrations and functional antibody activity. These results further indicate that there are significant differences depending on serotype in how ELISA-measured IgG levels relate to functional antibody activity.

### Vaccine status based on thresholds used

The variable distribution of IgG concentrations between serotypes (already observed by Park et al. [12]) has not been taken into account by the WHO or the American Academy of Allergy, Asthma & Immunology (AAAAI) guidelines recommending a single threshold of 1 or 1.3 µg/mL, respectively. This prompted us to investigate whether the heterogeneous distribution of IgG concentrations across serotypes could affect the interpretation of vaccine protection. We therefore compared patient vaccine response classification using the WHO and AAAAI fixed thresholds with classifications based on the serotype-specific PICs and the 95th percentile of the bootstrap-derived PICs while maintaining the criterion of ≥70% responder serotypes (Table 5).

**TABLE 5:** Diagnostic of protection according to the threshold of IgG concentration

Threshold of IgG concentrations		Number of patients with a diagnosis (%)	
		In favor of protection	Not in favor of protection
PIC		229 (31.41%)	500 (68.59%)
95th percentile PIC		160 (21.95%)	569 (78.05%)
WHO		412 (56.52%)	317 (43.48%)
AAAAI		325 (44.58%)	404 (55.42%)

Diagnosis is in favor of protection (according to the applied serological criterion) when at least 70% of serotypes are greater than or equal to the corresponding threshold; i.e., 0 to 4 responder serotypes for the group “not in favor of protection” and 5 to 7 responder serotypes for the group “in favor of protection.” IgG concentration thresholds are 1 µg/mL for the WHO and 1.3 µg/mL for the AAAAI. Pairwise McNemar tests with Holm correction demonstrated adjusted *P* values < 0.0001 for all comparisons.

For patients with 0 to 4 responder serotypes, results were not in favor of protection, whereas for patients with 5 to 7 responder serotypes, results were in favor of protection. Compared with the WHO and AAAAI fixed thresholds, the use of serotype-specific PICs classified fewer patients as having results in favor of protection. Using the serotype-specific PICs, 229 of 729 patients were classified as having results in favor of protection, compared with 412 of 729 using the WHO threshold and 325 of 729 using the AAAAI threshold. Using the 95th percentile of the bootstrap-derived PICs as a conservative sensitivity analysis further reduced this number to 160 of 729 patients. Cochran’s Q test on matched proportions demonstrated a significant difference in the number of patients considered “in favor of protective vaccine immunity” according to the threshold applied [Q(3) = 500.33, *P* < 0.0001, where Q corresponds to Cochran’s Q statistic with 3 degrees of freedom]. We then performed pairwise McNemar tests with Holm correction, which demonstrated highly significant differences between all thresholding strategies (all adjusted *P* < 0.0001), indicating that accounting for serotype-specific heterogeneity substantially alters the interpretation of pneumococcal vaccine responses.

## DISCUSSION

Addressing the threat of IPDs in high-risk patients, clinicians need to know, at a given time, whether their patients are protected against this pathogen. This guides decisions regarding vaccination or revaccination. The assessment of the protection status is carried out in our medical biology laboratory for seven vaccine serotypes according to the methods recommended by the WHO: the serotype-specific ELISA as a first-line assay and the OPA as a complementary test on a case-by-case basis.

A universal protective threshold has been defined for ELISA by the WHO and the AAAAI. However, there is serotypic heterogeneity before and after vaccination in healthy adults (11, 12). Our study also highlights heterogeneity between the seven serotypes tested in adults at risk of IPD. One of the causes of serotypic heterogeneity and immunogenicity is the structure of the polysaccharide capsule, which has an impact on recognition by the immune system, as shown for serotype 14 (16).

By analyzing specific IgG concentrations based on the binary classification of the OPA, we determined a PIC for each serotype that best predicted functional antibody activity in serum samples. This concentration, determined using ROC curves, represented a compromise between sensitivity and specificity for predicting OPA positivity. The AUC values ranged from 0.73 to 0.87, indicating satisfactory diagnostic performance. Analysis of the distribution of specific IgG concentrations between patients with positive OPA and patients with negative OPA highlighted heterogeneity in the immune response depending on the vaccine serotype studied. This heterogeneity was statistically confirmed by dispersion analyses of PIC calculated using the bootstrap method. PIC are lower than the serotype-specific thresholds established by Park et al*.* (12), which corresponded to vaccine response thresholds, to track the dynamics of the response before and after vaccination in healthy individuals. However, PIC are higher than 1 µg/mL (WHO cut-off) for six of the seven serotypes tested and higher than 1.3 µg/mL (AAAAI cut-off) for five of the seven serotypes tested, which reflects that IgG concentration is not always correlated to functionality.

Several serotypes displayed moderate sensitivity despite satisfactory overall diagnostic performance. In clinical practice, lower sensitivity may lead to underestimation of functional antibody activity for a subset of patients, whereas higher specificity limits false positive interpretation of vaccine protection. Therefore, PIC values should be considered as serotype-specific predictive IgG concentrations rather than definitive clinical protective thresholds.

In daily clinical practice, we evaluated the seven PCV-7 serotypes. The diagnosis is in favor of protection if at least five serotypes are above the corresponding cut-off concentration. However, for some patients with three or four responder serotypes, considering serotype heterogeneity, it is more difficult to conclude. The serotype-specific PIC may help refine the interpretation for these patients, particularly when OPA analysis cannot be performed.

This study has several limitations. First, clinical data are missing. We have no information about the clinical context of the patients, which prevents demographic or patient subgroup analyses. It also prevents us from determining seroconversion because we do not know the vaccination status. Our cohort reflects routine laboratory practice for pneumococcal vaccine response assessment. However, because clinical information was unavailable, external validation in well-characterized patient cohorts remains necessary before these PICs can be considered for broader clinical application. Second, the study only included adult patients. PICs determined for adults will not necessarily be similar to those found in the pediatric population, which is particularly at risk for IPDs and is one of the most important targets of vaccination campaigns. The marked serotype-dependent heterogeneity observed in ELISA IgG concentrations and their quantitative relationship with OPA titers questions the use of a single universal ELISA threshold for interpreting pneumococcal vaccine responses. Determining PIC using Youden indices and the bootstrap method highlighted the heterogeneity of the immune response against pneumococcal vaccine serotypes. The calculation of AUC with values ranging from 0.73 to 0.87 demonstrated satisfactory performance, leading us to consider the PIC as serotype-specific predictive concentrations for evaluating the pneumococcal vaccine response. Although more complicated to perform technically than ELISA, the OPA test remains indispensable and cannot be replaced, highlighting the complementarity of both tests. The heterogeneity of the immune response depending on the serotype raises questions about the relevance of a single threshold. Rather than determining a single threshold, taking into account the heterogeneity of the immune response depending on the serotype could be a valuable approach to improve the interpretation of pneumococcal vaccine responses. Future studies including external validation cohorts and additional pneumococcal serotypes will be required to confirm the applicability of this approach to routine interpretation of pneumococcal vaccine responses.

## Data Availability

The data sets generated and/or analyzed during the current study are available from the corresponding author upon reasonable request.

## SUPPLEMENTAL MATERIAL

Supplemental tables spectrum.00661-26-s0001.docx Tables S1 to S3.

ASM does not own the copyrights to Supplemental Material that may be linked to, or accessed through, an article. The authors have granted ASM a non-exclusive, world-wide license to publish the Supplemental Material files. Please contact the corresponding author directly for reuse.
