# Supplementary material for: Accounting for serotypic heterogeneity improves the interpretation of pneumococcal vaccine response using serotype-specific ELISA IgG concentrations
Source: Microbiol Spectr. 2026 Sep 11;14(10):e00661-26. doi: 10.1128/spectrum.00661-26 (PMC13689387; doi:10.1128/spectrum.00661-26)
Supplement: Supplementary file 1 [file spectrum.00661-26-s0001.docx]

Supplementary Table S1. Spearman correlation between serotype-specific ELISA IgG concentrations and OPA titers.

| **PsPN** | **Total** | **Spearman rho** | **Spearman p-value** | **Spearman test** |
| --- | --- | --- | --- | --- |
| 4 | 729 | 0.452 | <0.0001 | Two-sided Spearman rank correlation |
| 6B | 729 | 0.520 | <0.0001 | Two-sided Spearman rank correlation |
| 9V | 729 | 0.506 | <0.0001 | Two-sided Spearman rank correlation |
| 14 | 729 | 0.758 | <0.0001 | Two-sided Spearman rank correlation |
| 18C | 729 | 0.645 | <0.0001 | Two-sided Spearman rank correlation |
| 19F | 729 | 0.530 | <0.0001 | Two-sided Spearman rank correlation |
| 23F | 729 | 0.654 | <0.0001 | Two-sided Spearman rank correlation |

Spearman’s rank correlation coefficient (rho) was calculated using paired ELISA IgG concentrations and OPA titers (n=729) for each pneumococcal polysaccharide (PsPN). Correlations were two-sided and tested against the null hypothesis of rho=0.

Supplementary Table S2. Linear regression analysis describing the quantitative relationship between ELISA IgG concentrations and OPA titers.

| **PsPN** | **Valid paired** | **Regression slope** | **Regression intercept** | **Power equation coefficient** | **Regressionp-value** | **Log10 regression equation** | **Power regression equation** |
| --- | --- | --- | --- | --- | --- | --- | --- |
| 4 | 729 | 0.99 | 1.59 | 39.285 | <0.0001 | log10(OPA Titer) = 1.594228 + 0.994285 × log10(ELISA) | OPA Titer = 39.285143 × ELISA^0.994285 |
| 6B | 729 | 1.07 | 1.61 | 40.652 | <0.0001 | log10(OPA Titer) = 1.609086 + 1.070136 × log10(ELISA) | OPA Titer = 40.652378 × ELISA^1.070136 |
| 9V | 729 | 0.85 | 1.67 | 46.543 | <0.0001 | log10(OPA Titer) = 1.667858 + 0.853392 × log10(ELISA) | OPA Titer = 46.543344 × ELISA^0.853392 |
| 14 | 729 | 1.31 | 1.19 | 15.477 | <0.0001 | log10(OPA Titer) = 1.189674 + 1.308310 × log10(ELISA) | OPA Titer = 15.476532 × ELISA^1.308310 |
| 18C | 729 | 1.23 | 1.52 | 33.445 | <0.0001 | log10(OPA Titer) = 1.524325 + 1.226617 × log10(ELISA) | OPA Titer = 33.444544 × ELISA^1.226617 |
| 19F | 729 | 1.03 | 1.47 | 29.489 | <0.0001 | log10(OPA Titer) = 1.469665 + 1.025115 × log10(ELISA) | OPA Titer = 29.489323 × ELISA^1.025115 |
| 23F | 729 | 1.22 | 1.41 | 25.763 | <0.0001 | log10(OPA Titer) = 1.410993 + 1.221452 × log10(ELISA) | OPA Titer = 25.762782 × ELISA^1.221452 |

Linear regression was performed after log10-transforming ELISA IgG concentrations and OPA titers. Regression slope, intercept, regression p-value, and the corresponding equations on the log10 and original scales are presented for each PsPN.

Supplementary Table S3. Diagnostic performance of serotype-specific predictive IgG concentrations (PIC) for predicting OPA positivity.

| **PsPN** | **Valid paired** | **TP** | **FP** | **FN** | **TN** | **Sensitivity** | **Specificity** | **PPV** | **NPV** | **Accuracy** |
| --- | --- | --- | --- | --- | --- | --- | --- | --- | --- | --- |
| 4 | 729 | 220 | 90 | 154 | 265 | 0.588 | 0.746 | 0.710 | 0.632 | 0.665 |
| 6B | 729 | 205 | 47 | 191 | 286 | 0.518 | 0.859 | 0.813 | 0.600 | 0.674 |
| 9V | 729 | 241 | 82 | 133 | 273 | 0.644 | 0.769 | 0.746 | 0.672 | 0.705 |
| 14 | 729 | 321 | 66 | 77 | 265 | 0.807 | 0.801 | 0.829 | 0.775 | 0.804 |
| 18C | 729 | 285 | 70 | 111 | 263 | 0.720 | 0.790 | 0.803 | 0.703 | 0.752 |
| 19F | 729 | 311 | 48 | 193 | 177 | 0.617 | 0.787 | 0.866 | 0.478 | 0.669 |
| 23F | 729 | 229 | 55 | 94 | 351 | 0.709 | 0.865 | 0.806 | 0.789 | 0.796 |

Diagnostic performance was evaluated using paired ELISA and OPA measurements (n=729 for each serotype). OPA positivity served as the reference classification and serotype-specific predictive IgG concentrations (PIC) were determined using ROC curve analysis and Youden’s index.

TP: true positive; FP: false positive; FN: false negative; TN: true negative; PPV: positive predictive value; NPV: negative predictive value
